# Gut-derived β-amyloid: Likely a centerpiece of the gut–brain axis contributing to Alzheimer’s pathogenesis

**DOI:** 10.1080/19490976.2023.2167172

**Published:** 2023-01-22

**Authors:** Jinghua Jin, Zhi Xu, Lina Zhang, Can Zhang, Xiaoduo Zhao, Yuxuan Mao, Haojian Zhang, Xingguang Liang, Juanli Wu, Ying Yang, Jing Zhang

**Affiliations:** a Department of Pathology, the First Affiliated Hospital of Zhejiang University School of Medicine, Hangzhou, China; b Department of Neurobiology, NHC and CAMS Key Laboratory of Medical Neurobiology, School of Brain Science and Brain Medicine, and MOE Frontier Science Center for Brain Science and Brain-machine Integration, Zhejiang University School of Medicine, Hangzhou, China; c Central Laboratory, The First Affiliated Hospital of Zhejiang University School of Medicine, Hangzhou, China; d National Human Brain Bank for Health and Disease, The First Affiliated Hospital of Zhejiang University School of Medicine, Hangzhou, China

**Keywords:** β-amyloid, gut microbiota, gut–brain axis, Alzheimer’s disease, aging, cognition

## Abstract

Peripheral β-amyloid (Aβ), including those contained in the gut, may contribute to the formation of Aβ plaques in the brain, and gut microbiota appears to exert an impact on Alzheimer’s disease (AD) via the gut-brain axis, although detailed mechanisms are not clearly defined. The current study focused on uncovering the potential interactions among gut-derived Aβ in aging, gut microbiota, and AD pathogenesis. To achieve this goal, the expression levels of Aβ and several key proteins involved in Aβ metabolism were initially assessed in mouse gut, with key results confirmed in human tissue. The results demonstrated that a high level of Aβ was detected throughout the gut in both mice and human, and gut Aβ42 increased with age in wild type and mutant amyloid precursor protein/presenilin 1 (APP/PS1) mice. Next, the gut microbiome of mice was characterized by 16S rRNA sequencing, and we found the gut microbiome altered significantly in aged APP/PS1 mice and fecal microbiota transplantation (FMT) of aged APP/PS1 mice increased gut BACE1 and Aβ42 levels. Intra-intestinal injection of isotope or fluorescence labeled Aβ combined with vagotomy was also performed to investigate the transmission of Aβ from gut to brain. The data showed that, in aged mice, the gut Aβ42 was transported to the brain mainly via blood rather than the vagal nerve. Furthermore, FMT of APP/PS1 mice induced neuroinflammation, a phenotype that mimics early AD pathology. Taken together, this study suggests that the gut is likely a critical source of Aβ in the brain, and gut microbiota can further upregulate gut Aβ production, thereby potentially contributing to AD pathogenesis.

## Introduction

Alzheimer’s disease (AD) is the most common neurodegenerative disorder, accounting for 60-70% of dementia globally,^1,2^ and its prevalence as well as related social and economic issues are increasing exponentially as baby-boomers reach retirement age.^3^ Clinically, AD manifests as worsening impairment in memory and cognition, resulting in a gradual decline in mental, behavioral, and functional activities as well as a substantial decrease in the quality of daily life of the patients.^4,5^ Among more than 20,000 molecules screened for AD treatment over the last few decades, Aducanumab was the only one approved by the FDA in 2021.^6^ One of the main reasons for the difficulty in AD treatment is the lack of understanding of the precise mechanisms underlying AD development and progression.^7,8^

Pathologically, AD brain exhibits two features, i.e., extracellular β-amyloid (Aβ) plaques and intraneuronal neurofibrillary tangles (NFTs), which are essential for definitive AD diagnosis.^9,10^ Aβ accumulation due to overproduction and/or failure of clearance is believed to be one of the critical events during AD development.^11,12^ Traditionally, Aβ is considered to be derived from the amyloidogenic processing pathway, which involves sequential cleavages of amyloid precursor protein (APP) by β-secretase (BACE1) and related enzymes.^13,14^ However, the exact source of Aβ in AD brain remains unclear, as increasing lines of evidence suggest that Aβ can also be derived from the periphery,^15,16^ and the peripheral Aβ can be transported to the brain through the blood–brain barrier (BBB) or vagal nerve.^17,18^ Thus, it is possible that the peripheral Aβ is an important source of brain Aβ plaques and contributes to the pathogenesis of AD.

Aside from the source of Aβ, the gut-brain axis has received ample attention recently along with the discovery that the gut microbiota (GM), trillions of bacteria, fungi, and viruses found in gastrointestinal (GI) track, play a pivotal role in human diseases, including AD.^19–21^ Particularly, recent clinical studies observed alterations in the composition of GM in AD patients compared with healthy controls,^22–24^ strongly supporting the involvement of GM in AD pathogenesis. Furthermore, colonization of germ-free mutant APP/presenilin 1 (APP/PS1) transgenic mice with GM derived from conventionally-raised APP/PS1 transgenic mice drastically increased the cerebral Aβ pathology compared to wild type (WT) and germ-free transgenic mice,^25^ signifying that GM may play a causative or contributory role in AD onset and progression. However, the precise regulatory mechanism of GM in AD pathogenesis remains to be characterized.

In this study, we tested the hypothesis that gut-derived Aβ constitutes an important source of Aβ plaques in the brain, and we further investigated the regulation of the production of gut-derived Aβ, especially in the context of aging and altered GM in AD. Our findings provide a novel insight into the role of GM in the progression of AD pathogenesis.

## Results

### Gut constitutes a significant source of Aβ

**Figure 1.**
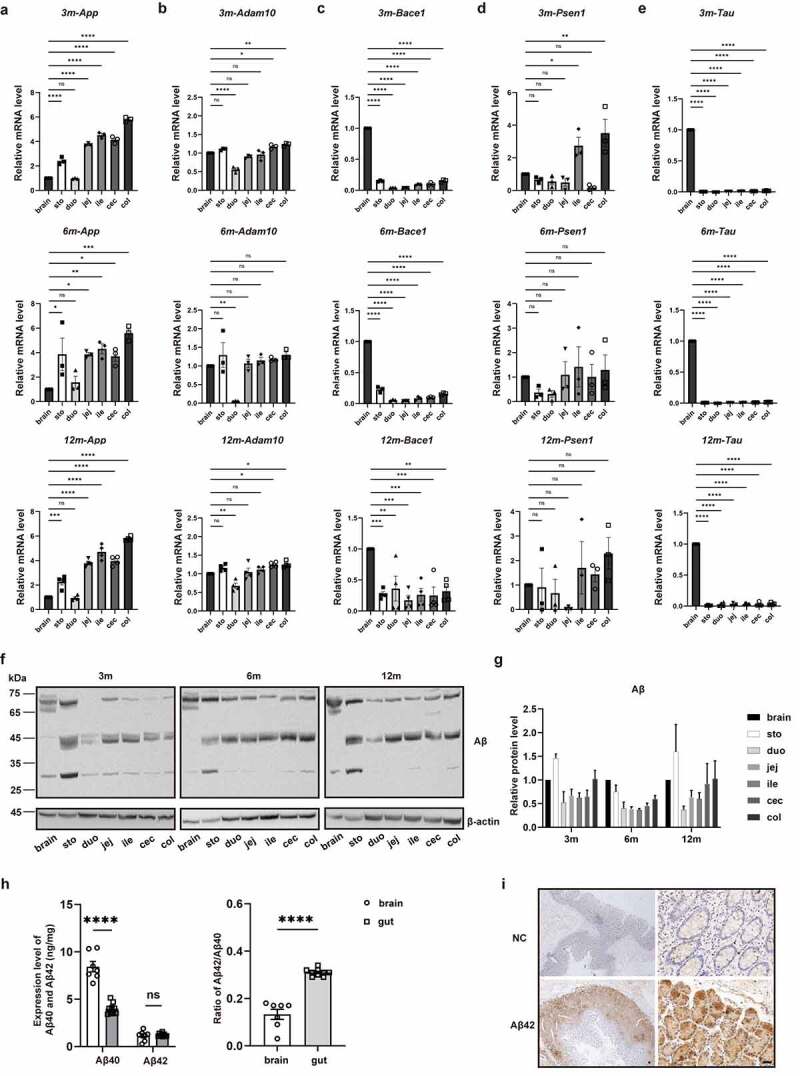
Gut is a significant source of Amyloid β. (a-e) Quantitative analysis of *App, Adam10, Bace1, Psen1* and *Tau* mRNA levels in 3-, 6-, and 12-month-old mice by qPCR (n = 3 or 4). Stomach (sto), duodenum (duo), jejunum (jej), ileum (ile), cecum (cec) and colon (col) were compared with brain for statistical significance. (f-g) WB images and quantitative analysis of Aβ protein level in 3-, 6-, and 12-month-old mice (n = 3) using anti-Aβ antibody (Invitrogen, H31L21, 700254). (h) Quantitative analysis of Aβ40/42 in human brain (n = 7) and gut (n = 9) tissues by Electrochemiluminescence immunoassay. (i) IHC images of Aβ42 stained by an anti-Aβ42 antibody (biolegend, 12F4, 805503) in human colon tissues. Scale bar = 100 μm. NC: negative control, staining with no primary antibody. The postmortem brain tissues were obtained from the China National Health and Disease Human Brain Tissue Resource Center, while the human intestinal tissues adjacent to cancer were obtained from patients at the First Affiliated Hospital of Zhejiang University School of Medicine. Detailed information was provided in Supplementary Table 1. Values are presented as means ± S.E.M, ordinary one-way ANOVA test for (a-e), and unpaired t test for (h). *, *P* < 0.05; **, *P* < 0.01; ***, *P* < 0.001; ****, *P* < 0.0001.

The expression levels of *App, Adam10, Bace1, Psen1, Psen2, Ncstn, Aph1a, Aph1b*, and *Tau*, genes closely linked to AD, were quantified using qPCR throughout the gut and brain of 3-, 6-, and 12-month-old mice. Compared to the brain, the mRNA level of *App* in the gut was notably higher, whereas the mRNA levels of *Bace1* and *Tau* were relatively lower in the gut. Yet, the levels of *Adam10, Psen1, Psen2, Ncstn, Aph1a, and Aph1b* showed no obvious difference (Figure 1a-e **& Supplementary Fig. 1**). The western blot (WB) analysis revealed that the Aβ was also expressed throughout the gut at a high level, with a few regions reaching the level close to that in brain tissue, regardless of age (figure 1f-g). The Aβ expression was further validated in human tissues with an electrochemiluminescence immunoassay. The results showed that Aβ42 and Aβ40 existed in the human gut and that the ratio of Aβ42/Aβ40 in the gut was significantly higher than that in the brain (Figure 1h, *P* < 0.0001). Additionally, immunohistochemical (IHC) staining of Aβ42 verified a strong positive staining in the epithelial cells of the human colon (Figure 1i), further suggesting that the gut is a significant source of Aβ.

## Gut Aβ42 increases with age in WT and APP/PS1 mice

**Figure 2.**
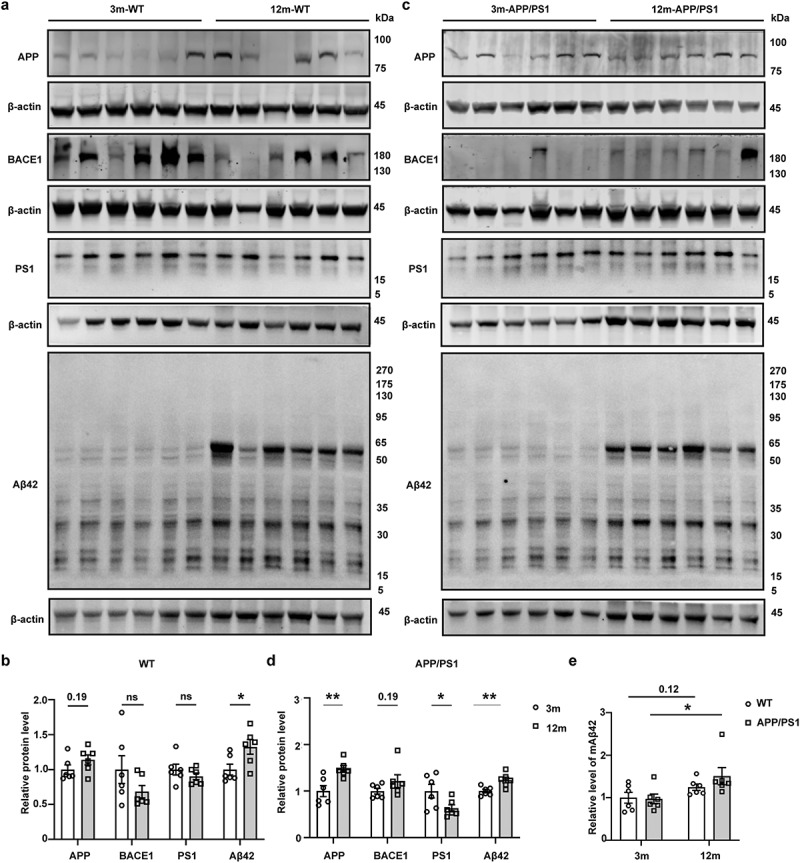
Gut Aβ42 increases with age in WT and APP/PS1 mice. (a & b) WB images and quantitative analysis of colonic APP (Invitrogen, CT695, 51-2700), BACE1 (Invitrogen, PA1-757), PS1 (Abcam, ab76083) and Aβ42 (biolegend, 12F4, 805503) protein levels in WT mice aged 3 (n = 6) and 12 months (n = 6). (c & d) WB images and quantitative analysis of colonic APP, BACE1, PS1, and Aβ42 protein levels in APP/PS1 mice aged 3 (n = 6) and 12 months (n = 6). (e) ELISA analysis of colonic mAβ42 protein level in WT and APP/PS1 mice aged 3 (n = 6) and 12 months (n = 6). Values are means ± S.E.M, unpaired t test. *, *P* < 0.05; **, *P* < 0.01.

Given that Aβ, especially Aβ42, is highly abundant in the gut compared with the brain at all ages, we next investigated whether the expression levels of gut APP, BACE1, γ-secretase subunit and Aβ42 are altered during the aging process in WT and APP/PS1 mice. Because of the high expression level of Aβ throughout the gut, a phenomenon also observed in the human colon, the colon was selected as a representative in all subsequent experiments. While the protein levels of BACE1, PS1 (Presenilin-1), PS2 (Presenilin-2) and Nicastrin in the colon exhibited no difference between WT mice at 3 and 12 months old (Figure 2a-b **& Supplementary Fig. 2A-B**), the level of APP showed a trend toward increase (Figure 2a-b, *P* = 0.19), and the protein level of Aβ42 was significantly higher in 12-month-old WT mice (Figure 2a-b, *P* < 0.05). In APP/PS1 mice, the expression levels of both APP and Aβ42 increased with age (Figure 2c-d, *P* < 0.01), the level of BACE1 showed a trend toward increase (Figure 2c-d, *P* = 0.19), while the levels of PS2 and Nicastrin showed no difference between APP/PS1 mice at 3 or 12 months old (**Supplementary Fig. 2C-D**). In fact, even a lower level of PS1 was detected in APP/PS1 mice at 12 months old (Figure 2c-d, *P* < 0.05). To confirm the observed higher level of Aβ42 in 12-month-old mice, samples were additionally blotted with 6E10, an antibody with a higher affinity to monomers, showing a similar trend (**Supplementary Fig. 2**). Furthermore, we compared the protein level of APP in colon of WT and APP/PS1 mice at 3 and 12 months old. The 12-month-old APP/PS1 mice had a higher level of APP compared with 3-month-old WT mice (**Supplementary Fig. 4A-C**, *P* < 0.05). Finally, the ELISA analysis of colonic Aβ42 protein level in WT and APP/PS1 mice aged 3 and 12 months confirmed the increase of Aβ42 with age in APP/PS1 mice (Figure 2e, *P* < 0.05 in APP/PS1 mice).

## Alteration of gut microbiota composition in aged APP/PS1 mice

**Figure 3.**
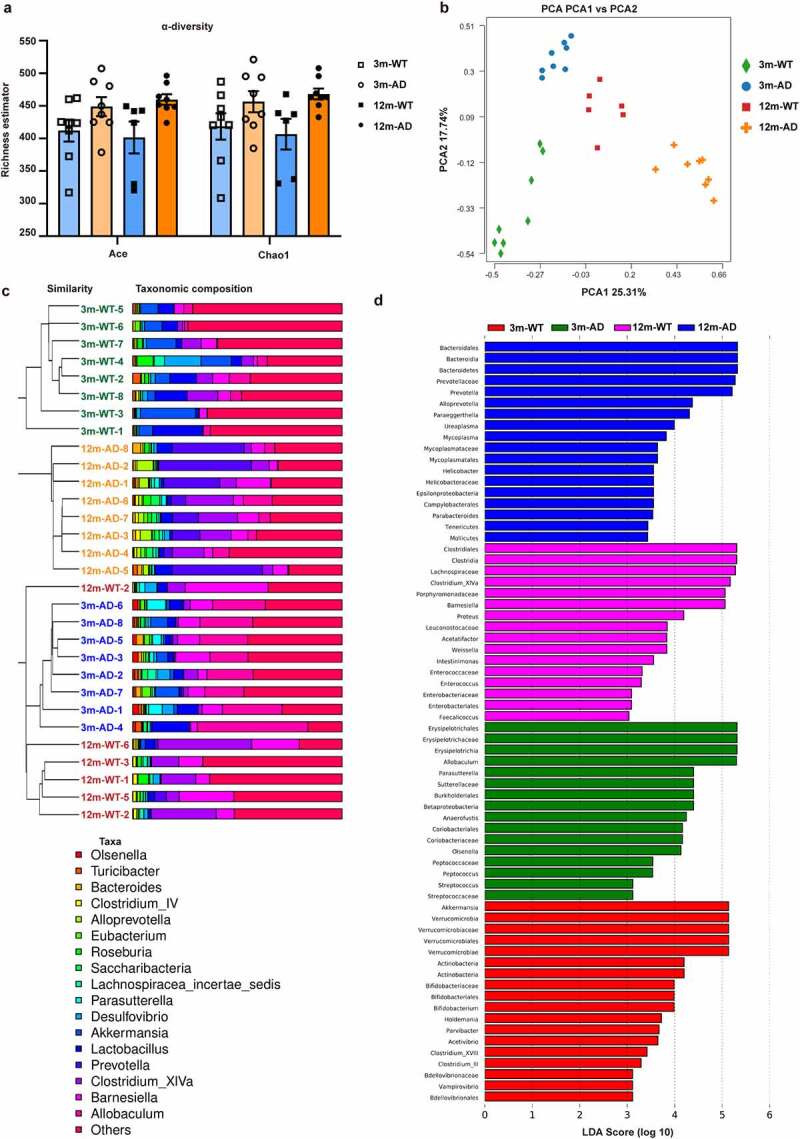
Alterations of gut microbiome in old APP/PS1 mice. (a) Ace richness estimator and Chao1 richness estimator, (b) PCA, (c) ClusterTree on genus with unweighted UniFrac dissimilarities, and (d) LefSe analysis of the 16S rRNA gene-sequencing dataset of feces from 3-month-old WT mice (3 m-WT, n = 8), 3-month-old APP/PS1 mice (3 m-AD, n = 8), 12-month-old WT mice (12 m-WT, n = 6), and 12-month-old APP/PS1 mice (12 m-AD, n = 8).

AD is clearly an age-related disease and increasing evidence demonstrates GM alterations with age, especially in AD patients.^26–29^ Therefore, we theorized that GM may be involved in the pathogenesis of AD by regulating gut-derived Aβ. To test this hypothesis, we first examined whether there were gut microbiome alterations in aged APP/PS1 mice. Feces from 3- and 12-month-old APP/PS1 mice as well as age-matched controls were collected for 16S rRNA sequencing. No significant differences were observed in the α-diversity, i.e., Ace richness estimator and Chao1 richness estimator, among the 3- and 12-month-old WT and APP/PS1 mice (Figure 3a), indicating that the total number of species of flora was similar in these mice. On the β-diversity, however, principal component analysis (PCA) (Figure 3b) and ClusterTree on genus with unweighted UniFrac dissimilarity analysis (Figure 3c) showed significant differences between 3- and 12-month-old mice, indicating that the composition of gut microbiota was altered with age. Besides, there were significant differences in the flora species between WT and APP/PS1 mice at each age compared. For example, the abundance of several floras including *Bacteroidales, Bacteroidia, Bacteroidetes, Prevotellaceae*, and *Prevotella* increased in 12-month-old APP/PS1 mice, as shown by the LefSe analysis (Figure 3d).

Previous studies have reported that a leaky and inflamed gut was closely related to gut microbiota composition.^30–32^ We further investigated, by FITC-dextran administration, whether the permeability of the gut was altered along with the composition change of gut microbiota in aged APP/PS1 mice. The results revealed that the permeability of the gut in older mice was higher than that of the young mice, with the 12-month-old APP/PS1 mice exhibiting the highest gut permeability (**Supplementary Fig. 4D**). Consistently, the abundance of pro-inflammatory bacteria, including the *Bacteroides*,^33^ *Prevotella*,^34^ and *Ruminococcus*,^35^ was significantly higher in the 12-month-old APP/PS1 mice, whereas the abundance of some beneficial gut bacteria, including the Akkermansia,^36^ Bifidobacterium,^37^ and Lactobacillus,^37^ decreased (**Supplementary Fig. 4E**).

## Fecal microbiota transplantation alters the levels of BACE1 and Aβ42 in gut

**Figure 4.**
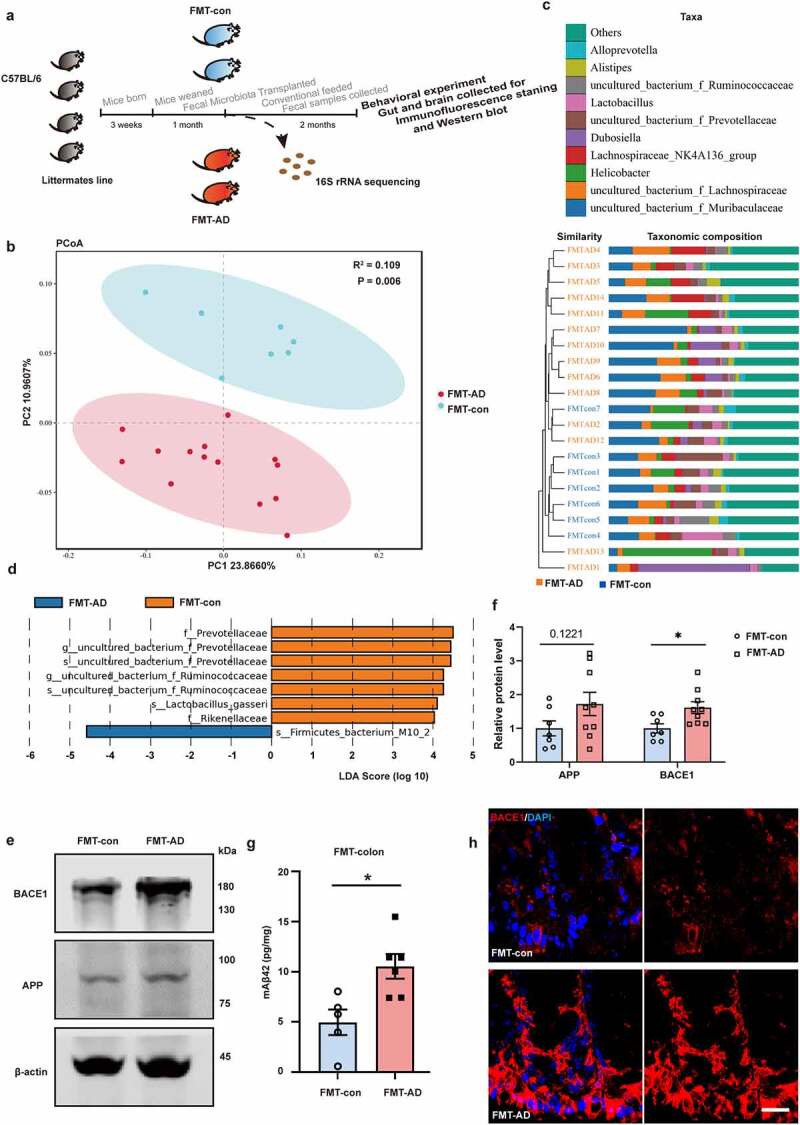
Fecal microbiota transplantation alters the gut microbiome and increases the levels of BACE1 and Aβ42 in gut of young WT mice. (a) Schematic diagram of fecal microbiota transplantation and follow-up experiments. Fecal microbiota of 12-month-old APP/PS1 mice were transplanted to 3-week-old WT mice that were just weaned (FMT-AD). 3-week-old littermates gavaged with their own fecal microbiota were served as control (FMT-con). (b) PCoA with binary-Jaccard UniFrac distances, (c) ClusterTree on genus with Bray-Curtis UniFrac dissimilarities, and (d) LefSe analysis from the 16S rRNA gene-sequencing dataset of FMT-con (n = 7) and FMT-AD (n = 14). (e & f) WB images and quantitative analysis of the BACE1 and APP protein levels in the colon of FMT-con (n = 7) and FMT-AD (n = 9). (g) Quantitative analysis of Aβ42 in colon tissues of FMT-con (n = 5) and FMT-AD (n = 6) by ELISA. (h) Confocal images of BACE1 in the colon of FMT-con and FMT-AD. Scale bar = 20 μm. Values are means ± S.E.M, unpaired t test. *, *P* < 0.05.

To further test whether GM from aged APP/PS1 mice would alter the production of gut Aβ, fecal microbiota of 12-month-old APP/PS1 mice were transplanted to 3-week-old WT mice that were just weaned. Three-week-old littermates gavaged with their own fecal microbiota were served as control (FMT-con). Feces were collected for 16S rRNA sequencing after 1 month of gavage in the FMT recipients (Figure 4a). According to the PCoA with binary-Jaccard UniFrac distances (Figure 4b) and ClusterTree on genus with Bray-Curtis UniFrac dissimilarities (Figure 4c), 1 month of FMT was able to alter the microbiota of young WT mice substantially. LefSe analysis gave a more comprehensive result of the differences at the levels of phylum, class, order, family, genus, and species (Figure 4d).

Regarding the effect of FMT on the Aβ metabolism, the levels of BACE1 and APP were analyzed by WB. The results showed that the expression level of colonic BACE1 was significantly higher in the WT mice transplanted with feces of APP/PS1 mice (FMT-AD) than that of FMT-con (Figure 4e & f, *P* < 0.05) and the expression level of APP showed an increasing trend although not statistically significant (Figure 4e & f, *P* = 0.1221). The increased level of colonic BACE1 in FMT-AD was further confirmed by immunofluorescent (IF) analysis (Figure 4h). Additionally, the ELISA analysis revealed that the level of Aβ42 in the colon increased significantly in FMT-AD compared with that of FMT-con (Figure 4g, *P* < 0.05).

## Transmission of gut Aβ42 to the brain is mainly via blood in aged mice

**Figure 5.**
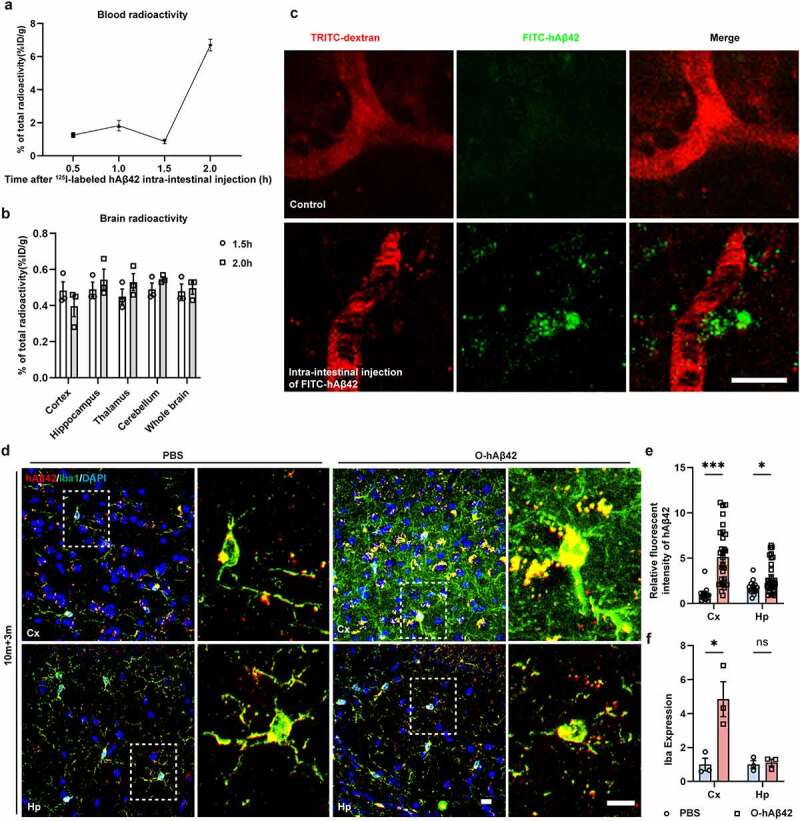
Intra-intestinal injected Aβ42 is readily transferred from the gut to the brain. (a & b) The radioactivity of blood and brain samples of 10-month-old mice after intra-intestinal injection of ^125^ I-labeled hAβ42. (c) Two-photon intravital images of intra-intestinal-injected FITC-labeled hAβ42 in the brain of 10-month-old WT mice. Scale bar = 10 μm. (d & f) Three-dimensional confocal images of hAβ42 (biolegend, 6E10, 803015, human specific) and microglia (anti-Iba1) and quantitative analysis of hAβ42 and Iba1 3 months after intra-intestinal injection of PBS or oligomers of hAβ42 (O-hAβ42) in 10-month-old mice (10 m + 3 m). Scale bar = 10 μm. Values are means ± S.E.M, unpaired t test. ns, no significance; *, *P* < 0.05; ***, *P* < 0.001.

Having confirmed that both aged APP/PS1 and FMT-AD mice exhibited a significantly higher level of gut Aβ42, we next examined whether the gut-derived Aβ could be transported into the brain. To test this, ^125^ I- or FITC-labeled hAβ42 (i.e., human Aβ42) peptides were injected into the gut of 10-month-old WT mice. The radioactivity of blood was detected time-dependently after intra-intestinal injection of ^125^ I-labeled hAβ42 peptides (Figure 5a). The radioactivity in the heart, liver, spleen, lung, and kidney suggested that the gut-injected Aβ can be transported to the whole body (**Supplementary Fig. 5E**). The radioactivity of the brain was detected at 1.5 h and 2 h after intra-intestinal injection, and there was no difference between different brain regions (Figure 5b, *P* > 0.05). Consistently, two-photon intravital imaging revealed that FITC-labeled hAβ42 peptides were identified in the brain parenchyma 2 hours after injection (Figure 5c), indicating that Aβ42 is readily transferred from the gut to the brain. To further support this observation, 10-month-old WT mice were injected with oligomeric hAβ42 (O-hAβ42) in the gut and then sacrificed 3 months later (10 m + 3 m). ELISA analysis of hAβ42 in the gut and plasma showed that the gut-derived injected Aβ can be detected in the blood in addition to the gut (**Supplementary Fig. 5 F & G**). The IF results further supported that the gut-injected O-hAβ42 was transported to the brain even after 3 months (**Supplementary Fig. 5 h**) and deposited in the cortex and the hippocampus, with most Aβ deposits seen in microglia (Figure 5d, e). Importantly, the microglia in the cortex of the mice with gut-injected O-hAβ42 were significantly activated compared with the vehicle-treated mice (Figure 5d, f, *P* < 0.05), suggesting that gut-injected O-hAβ42 can be transmitted to the brain and activate microglia potently.

**Figure 6.**
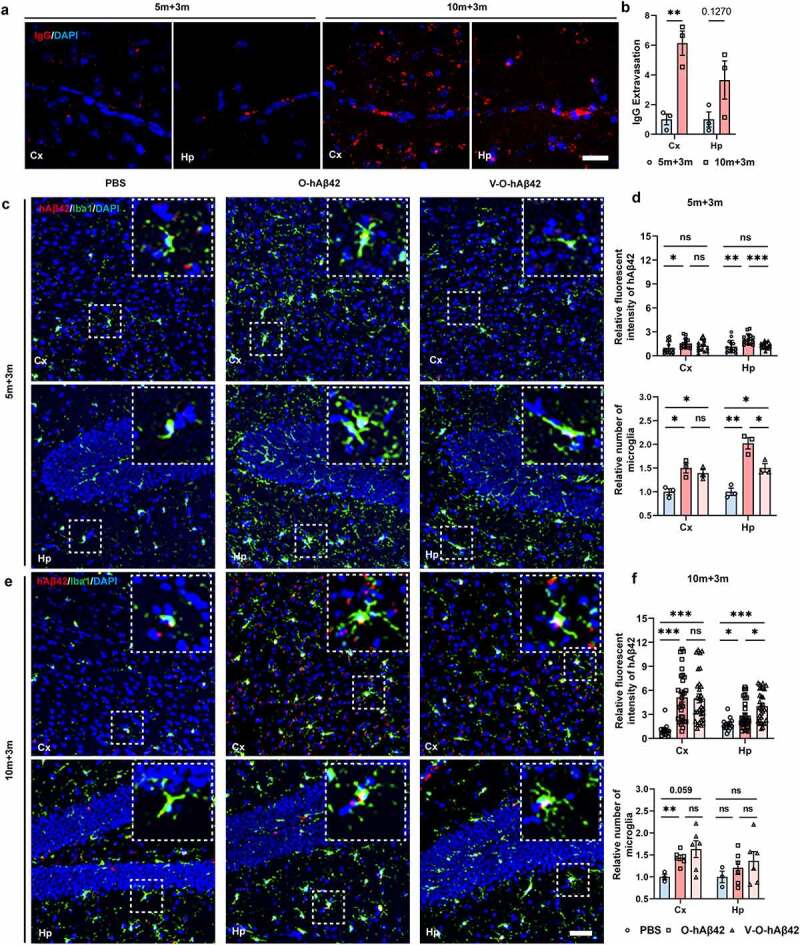
Transmission of gut Aβ42 to the brain is mainly via the blood in aged mice. (a & b) Three-dimensional confocal images and quantitative analysis of cerebral extravasated plasma IgG 3 months after intra-intestinal injection of PBS in 5-month-old (5 m + 3 m) and 10-month-old mice (10 m + 3 m). Scale bar = 20 μm. (c & d) Confocal images and quantitative analysis of hAβ42 (biolegend, 6E10, 803015, human specific) and microglia (anti-Iba1) 3 months after intra-intestinal injection of PBS or O-hAβ42 and vagotomy before intra-intestinal injection of oligo-hAβ42 (V-O-hAβ42) in 5-month-old WT mice. (e & f) Confocal images and quantitative analysis of hAβ42 and microglia (anti-Iba1) 3 months after intra-intestinal injection of PBS or O-hAβ42 and V-O-hAβ42 in 10-month-old WT mice. Scale bar = 50 μm. Values are means ± S.E.M, unpaired t test. ns, no significance; *, *P* < 0.05; **, *P* < 0.01; ***, *P* < 0.001.

Previous study demonstrated that peripheral Aβ42 can be transmitted from the enteric nervous system to the brain via the vagal nerve.^17^ However, others have suggested that blood Aβ42 can also be readily transmitted to the brain.^38,39^ To determine the relative contribution of these two routes in the transmission of O-hAβ42, gastric vagotomy^40^ was performed before the intra-intestinal injection of O-hAβ42 in 5- and 10-month-old mice (V-O-hAβ42), respectively, and the mice were sacrificed 3 months later. Compared with 5-month-old mice, 10-month-old mice had obviously increased brain extravasation of plasma-borne IgG in the cortex, an established marker of blood–brain barrier (BBB) dysfunction,^38^ indicating that the permeability of the BBB increased in older mice (Figure 6a, b, *P* < 0.01). Additionally, in 5-month-old mice, hAβ42 signals significantly increased both in the cortex (*P* < 0.05) and the hippocampus (*P* < 0.01) after intra-intestinal injection of O-hAβ42 compared with the vehicle-treated mice (Figure 6c & d). The deposition of gut-injected O-hAβ42 was also detected in the cortex and hippocampus in 10-month-old mice (Figure 6e & f). However, the IF signal of hAβ42 in the cortex and hippocampus of 5-month-old mice was much lower than that of 10-month-old mice after intra-intestinal injection of O-hAβ42 (Figure 6c-f), consistent with the observation of the BBB result discussed above. Notably, in 5-month-old mice with better preserved BBB integrity, the increasing hAβ42 signal in the hippocampus was largely abolished in mice with vagotomy (*P* < 0.001), suggesting the contribution of vagal nerves in the transmission of O-hAβ42 to hippocampus in 5-month-old mice. In contrast, in 10-month-old mice with more compromised BBB integrity, there were no obvious differences between mice with or without vagotomy in the cortex. In fact, the signals were even higher after vagotomy in the hippocampus compared to the group without vagotomy, indicating that the contribution of vagal nerve route in the transmission of O-hAβ42 largely decreased in 10-month-old mice. Consistently, gastric vagotomy significantly reduced microglial activation in the hippocampus of 5-month-old mice (Figure 6c, d, *P* < 0.05), but not in 10-month-old mice (Figure 6e & f). In short, the contribution of vagal nerves in the transmission of O-hAβ42 from the gut to the brain is age dependent and influenced substantially by the integrity of the BBB.

## FMT induces early Alzheimer’s pathology

**Figure 7.**
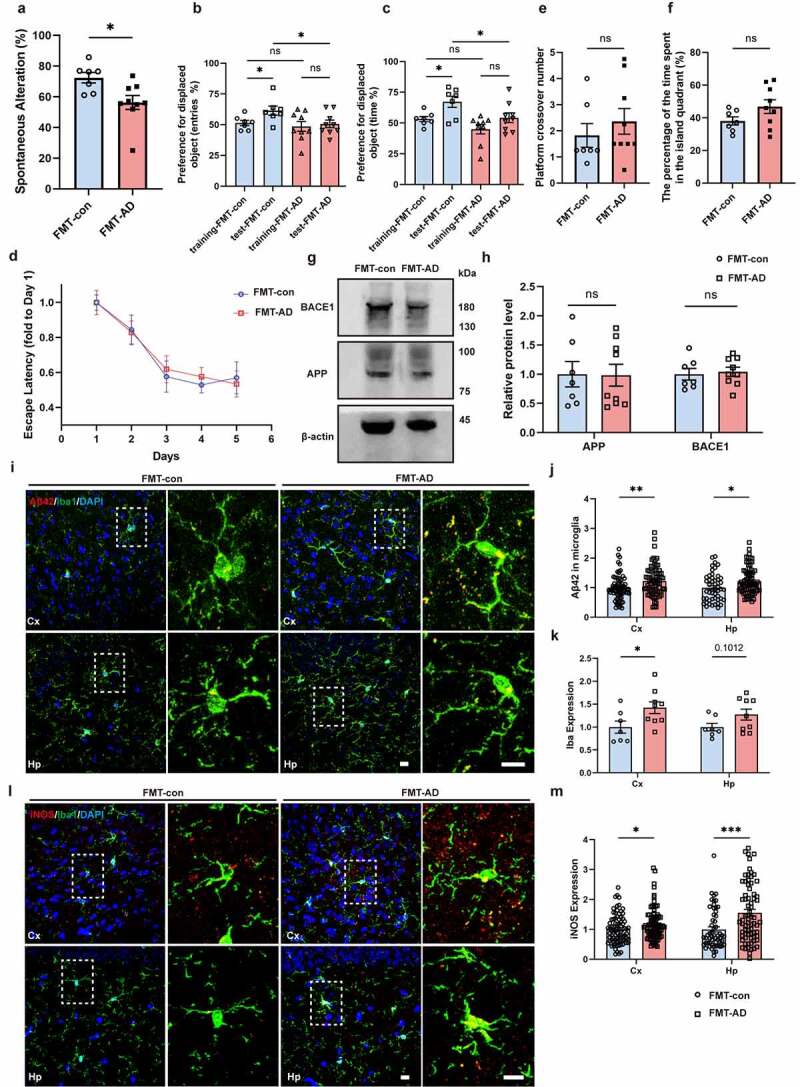
Fecal microbiota transplantation induces Alzheimer’s disease-like cognitive impairment and neuropathology. Y-maze (a), novel object recognition (b & c) and Morris’ water maze (d-f) analysis of 3-week-old WT mice gavaged with fecal microbiota from 12-month-old AD mice (FMT-AD, n = 9) and littermates gavaged with their own fecal microbiota as control (FMT-con, n = 7). (g & h) WB images and quantitative analysis of cerebral BACE1 and APP protein levels in FMT-con (n = 7) and FMT-AD (n = 9). (i-k) Three-dimensional confocal images and quantitative analysis of mAβ42 and microglia (anti-Iba1) in FMT-con (n = 7) and FMT-AD (n = 9). Scale bar = 10 μm. (l & m) Three-dimensional confocal images and quantitative analysis of iNOS in FMT-con (n = 7) and FMT-AD (n = 9). Scale bar = 10 μm. Values are means ± S.E.M, unpaired t test. ns, no significance; *, *P* < 0.05; **, *P* < 0.01; ***, *P* < 0.001.

Given the findings that FMT-AD increased the production of gut Aβ42 in young WT mice and gut Aβ entered the brain readily, we further explored the role of GM-induced gut Aβ in AD pathogenesis. Three behavioral tests were performed 2 months following FMT to evaluate the impact of GM on the mouse cognition, i.e., spontaneous Y-maze (spatial working and short-term memory), novel object recognition (memory), and Morris’ water maze (spatial working memory). Compared with FMT-con mice, FMT-AD mice scored lower on Y-maze (Figure 7a) and novel object recognition (Figure 7b & c) but showed similar performance on Morris’ water maze analysis (Figure 7d-f), indicating that the alterations of intestinal flora in WT mice were able to influence the short-term spatial memory and memory for novel object recognition of the mice, even at an early stage.

Following the behavioral tests, the brain tissues were collected for immunostaining and WB analysis. Neither obvious amyloid plaques nor different expressions of APP and BACE1 were detected in the brain (Figure 7g & h). However, higher levels of Iba1 and iNOS, two commonly used indicators of microglial activation, were observed in the cortex and the hippocampus of FMT-AD (Figure 7i-m). Most importantly, a higher level of Aβ42 in microglia was detected both in the cortex (Figure 7i, j, *P* < 0.01) and the hippocampus (Figure 7i, j, *P* < 0.05) of FMT-AD, suggesting that microglial activation induced by FMT of APP/PS1 mice increases the uptake of Aβ42 in the brain, especially in the microglia.

## Discussion

The major findings of this study include 1) the gut is a major source of Aβ peptides, with its production further enhanced by aging process and APP/PS1 mice-derived GM; 2) transmission of gut Aβ42 to the brain is mainly via blood, instead of the vagal nerve, in aged mice; 3) transplanting fecal bacteria of aged APP/PS1 mice can provoke functional and pathological alterations in young mice, mimicking early AD pathology.

Traditionally, Aβ is thought to be produced mainly by neurons via amyloidogenic processing of the APP in the brain. Nonetheless, several recent studies have shown that, besides the brain, the enterocytes also express Aβ,^41–43^ and cholesterol-rich diets can regulate the Aβ presence in the gut epithelium.^44^ In the current study, we found that the mRNA level of *APP* in the GI was 2-6 times higher than that in the brain. In contrast, the mRNA level of *Tau*, another gene/protein closely related to the pathogenesis of AD, is much lower in the GI than that in the brain. Thus, APP and its proteolytic production Aβ, instead of the Tau species, likely play a pivotal role in the periphery, especially in the GI tract, in terms of contribution to AD development and progression.

The function of peripheral Aβ remains elusive. Recently, Aβ has been recognized as an antimicrobial peptide and even as a part of the innate immune system.^45,46^ Compared to Aβ40, Aβ42 is less abundant but more toxic, and it forms the core of antimicrobial pore-forming structures^47^ in addition to being a key component of senile plaques in the AD brain. It has been suggested that the ratio of Aβ42/Aβ40, instead of the total Aβ level, determines the Aβ42-driven pathological pathway. Furthermore, a selective reduction of the Aβ42/Aβ40 ratio may provide a potential therapeutic strategy for AD treatment in the future.^48^ In this study, we showed that there was a relatively higher ratio of Aβ42/Aβ40 in the gut than in the brain of human. The higher ratio of Aβ42/Aβ40 in the human gastrointestinal (GI) track further indicates its potential contribution to brain pathology of AD besides regulating GM of the GI track. One caveat in our study is that the brain and gut tissue were collected from different donors, making a direct correlation of the levels of Aβ between the brain and gut for each of these individuals impossible.

How might gut Aβ42 species be transported to the brain is a matter of controversy. It has been reported that the enteric Aβ administration directly induces AD-like dementia and cerebral amyloidosis, which may be induced by a retrograde axonal transportation via the vagus.^18^ However, others argue that a blood route of transportation might be equally important.^16,39^ To further characterize the pathways for gut-derived Aβ to enter the brain, we compared the contribution of blood circulation versus vagal nerve in young and old mice. Our data suggest that both blood route and vagal nerve contribute to the transmission of intestinal Aβ into the brain; however, the relative contributions of each route are age dependent. It should be noted that, in addition to Aβ transportation in AD pathology, the gut-to-brain spreading of other prion-like proteins, e.g., α-synuclein, have also been studied in mice, rats and baboon monkeys,^40,49–51^ suggesting that the gut is likely important in other neurodegenerative disorders.

As indicated earlier, GM were altered in AD patients^52–55^ and rodent AD models.^30,31,56^ Indeed, dysbiosis and alterations of the GM composition have been observed in several human diseases, including AD, leading to a concept of gut–brain axis in recent years.^57–59^ Clearly, the composition of GM can affect the ability of cognition, learning, and memory in rodents^30,56^ as well as in humans.^53,54^ However, the mechanisms underlying AD-related gut-brain axis remain to be fully defined. Several investigators believe that GM may affect the central nervous system via the vagal nerve, signaling mediators of the immune system, enteric hormones, and GM-derived metabolites.^58–60^ Our investigation suggests that gut Aβ42 might be another major driver that integrates aging, GM, and AD progression together. We sequenced the bacterial 16S rRNA from fecal samples of aged APP/PS1 mice and found a remarkable shift in the GM composition compared to young WT mice. The FMT of APP/PS1 mice significantly increased the levels of BACE1 and Aβ42 in the colon of WT mice, suggesting that GM might regulate the Aβ production in the gut. Notably, although Aβ from gavaged feces may also be absorbed by the gut and contribute to the Aβ pathology in the brain, very little Aβ was detected in the fecal gavage supernatant in the experimental conditions in the study (**Supplementary Fig. 5I**).

Previous studies have shown that transplantation of healthy microbiota reduces amyloid and tau pathology in an AD model^30^ as well as counteracts with selective age-associated behavioral deficits even in WT mice.^61^ In our study, transplanting fecal microbiota of aged APP/PS1 mice readily provoked functional and pathological alterations, simulating AD-like cognitive impairment and microglial activation in young WT mice, a stage typically showing no Aβ deposition or functional impairment even in APP/PS1 mice.^62^ Increased microglial activation, with engulfed intracellular Aβ, was observed in FMT-AD in the absence of Aβ plaques, indicating that gut-derived Aβ might be quite crucial in early AD development by increasing neuroinflammation in the brain. Indeed, a previous study reported that the inflammation of the gut may promote the formation of Aβ fibrils in the gut, further resulting in synaptic degeneration and impaired associative memory of mice.^17^ Moreover, several population cohort studies revealed that the patients with inflammatory bowel disease^63^ or individuals with higher pro-inflammatory-potential diets^64^ were associated with a higher risk of dementia. Supplementation of pro-/symbiotic bacteria, on the other hand, is likely to increase the level of brain-derived neurotrophic factors, protecting neuronal function.^65^ Nonetheless, the precise mechanisms involved in the AD pathology of FMT-AD in mouse brain, including inflammatory, need to be investigated further. In future studies, it is also necessary to study the effect of GM on the Aβ deposition in the brain in a longer-term longitudinal investigation of WT and APP/PS1 mice.

In summary, our study suggests that the gut is likely a critical source of Aβ in the brain, and gut Aβ increased with age in WT and APP/PS1 mice. Additionally, alternations of GM further upregulated the production of gut Aβ, possibly contributing to the pathogenesis of AD. The orchestrated relationship among gut Aβ, aging, and GM suggests a potentially novel therapeutic approach for the treatment of AD by modulating GM, specifically via personalized diet or probiotic intervention to regulate gut Aβ metabolism.

## Patients and Methods

### Human samples

This study was approved by the Institutional Review Board of First Affiliated Hospital of Zhejiang University School of Medicine, Hangzhou, Zhejiang, China (IIT20220143B). The postmortem brain tissues were obtained from the China National Health and Disease Human Brain Tissue Resource Center (Hangzhou, China). All materials were collected from donors who had provided written informed consent for a brain autopsy and permitted their clinical information for research purposes. The human intestinal tissues adjacent to cancer were obtained from the patients at the First Affiliated Hospital of Zhejiang University School of Medicine who provided written informed consent. A summary of the demographics and clinical data of the participants is provided in **Supplementary Table 1**.

## Animals and antibodies

All animal studies and experimental procedures were approved by the Animal Care and Use Committee of the animal facility at Zhejiang University (20221077). The WT mice (C57BL/6) and APP/PS1 mice were purchased from Hangzhou Ziyuan Biotechnology Co., LTD. The mice were housed on a 12-h light–dark cycle with free access to food and water.

The following antibodies were used for western blot (WB), immunohistochemical (IHC) staining, and immunofluorescence (IF) staining: mouse monoclonal [AC-74] to β-actin (A5316, Sigma) for WB; rabbit monoclonal [H31L21] to Aβ (700254, Invitrogen) for WB and Dot blot; rabbit polyclonal [CT695] to APP (51-2700, Invitrogen) for WB and Dot blot; rabbit polyclonal antibody to BACE1 (PA1-757, Invitrogen) for WB; mouse monoclonal [12F4] to Aβ, 1-42 (805503, Biolegend) for WB, IHC, and IF; Rabbit monoclonal [EP2000Y] to PS1 (ab76083, Abcam) for WB; rabbit polyclonal to PS2 (16168-1-AP, Proteintech) for WB; rabbit polyclonal to Nicastrin (14071-1-AP, Proteintech) for WB; mouse monoclonal [6E10] to human Aβ, 1-16 (803015, Biolegend) for IF and WB; rabbit polyclonal to oligomers (AHB0052, Invitrogen) for Dot blot; rabbit polyclonal antibody to Iba1 (019-19741, Wako) for IF; rat monoclonal [RMG1-1] to mouse IgG1 (406602, Biolegend) for IF. Homemade dye AH-2 was used to confirm the oligomerization of hAβ42. The horseradish peroxidase (HRP)-conjugated secondary antibodies used in WB and the Alexa Fluor 565 and 633 conjugated secondary antibodies used in IF were purchased from Thermo Fisher Scientific. Dylight 594 goat anti-mouse conjugated secondary antibodies used in IF were purchased from Earthox. Goat anti-mouse IRDye® 800CW conjugated secondary antibody (926-32210) and donkey anti-rabbit IRDye® 800CW conjugated secondary antibody (926-32213) used in WB were purchased from Odyssey. The antibodies used to detect APP or Aβ were validated in **Supplementary Fig. 3**.

## Quantitative real-Time-PCR (qPCR)

The quantitative Real-Time-PCR analysis was performed according to the instructions (Q711, Vazyme). The primers for mRNAs are listed in **Supplementary Table 2**.

## Western blot

Tissues were homogenized, and the protein concentration was determined through the Protein Quantification Kit (23225, Thermo Fisher Scientific). Samples were loaded onto gradient gels (C35552111, GenScript) and transferred onto a nitrocellulose blotting membrane (T500361, PALL). After blocking, the membranes were incubated with the primary antibody followed by incubation with an HRP-conjugated secondary antibody or IRDye® 800CW conjugated secondary antibody. Images were visualized using a ChemiDoc MP imaging system (Bio-Rad). The results were analyzed using ImageJ software with β-actin as internal controls. Full-length Western blots were shown in supplementary materials.

## Electrochemiluminescence immunoassay for detection of Aβ40 and Aβ42

The human tissues were cut into small pieces, homogenized, and ultrasonically cracked under the conditions of power: 300 W, ultrasonic time: 2 s, interval time: 15 s on ice for 5 times. Then, the samples were centrifuged, and the supernatant was transferred to a clean tube. The concentration of Aβ40 and Aβ42 was then determined by a homebrew kit (Beijing Xystarneu Biotechnology Co., Ltd.) on a chemiluminescence analyzer (eCL8000).

## Immunohistochemical staining

The human colon sections were dewaxed, rehydrated, and rinsed in distilled water. After incubated with 1% hydrogen peroxide and sodium citrate buffer, the slides were permeabilized with blocking buffer (1% BSA, 0.03% Triton X-100, and 4% NGS in PBS), incubated with anti-Aβ42 (805503, Biolegend) and biotinylated secondary antibody (PV6002, Beijing Zhongshan Golden Bridge Biotechnology Co., Ltd.) sequentially, and reacted with 3,3ʹ-Diaminobenzidine chromogenic solution (ZLI-9018, Beijing Zhongshan Golden Bridge Biotechnology Co., Ltd.). Later, the slides were counterstained in Mayer’s hematoxylin, rinsed in water before dehydration in 65% ethanol, 75% ethanol, 80% ethanol, 90% ethanol, 100% ethanol, and 2× xylene, and mounted with DPX Mountant (MM1410-100ML, MKbio).

## Aβ42 ELISA

The protein of colon was extracted and determined through Protein Quantification Kit (23225, Thermo Fisher Scientific). The levels of injected hAβ42 in the colon tissues and plasma of mice were determined by human Aβ42 ELISA Kit (KHB3544, Invitrogen). The levels of mAβ42 in the colon tissues of mice were determined by mouse Aβ42 ELISA Kit (ml002201V, mlbio, and KMB3441, Invitrogen).

## *In vivo* permeability assay

*In vivo* intestinal permeability was measured by the FITC-dextran assay. Mice were starved for 6 h prior to the start of the experiments. FITC-dextran (46944, Sigma Aldrich) was orally gavaged at a dosage of 400 mg/kg. Blood samples were collected with the serum measured at an excitation wavelength of 490 nm and an emission wavelength of 530 nm using a spectrofluorometer. FITC-dextran diluted in PBS was used to plot a standard curve, and the serum concentration of FITC-dextran was calculated.

## ^125^ I-Aβ42 labeling

The Human Aβ42 (hAβ42) (A834109, Shanghai Macklin Biochemical Technology) was reacted with 1 mCi of ^125^ I in an Iodogen tube, and the mixture was loaded onto a C18 Sep-Pak cartridge (Waters Corporation) after using methanol and ultrapure water for activation. After the removal of free ^125^ I ions, the labeled product was eluted by ethanol, dried by nitrogen and reconstituted by DMSO. The radiochemical purity was >95% as determined by paper chromatography. hAβ42 with ^125^ I was injected into the intestine of the mice. The radioactivity of organs, blood, and brain was detected by an automatic γ detector.

## Oligomerization

The oligomeric hAβ42 was prepared as previously reported.^66^ In brief, monomeric hAβ42 was incubated at 4°C for 48 h with stirring, and the oligomerization was confirmed by dot blot (**Supplementary Fig. 5D**) as well as immunostaining in the brain by a homemade oligomer detection dye, AH-2 (**Supplementary Fig. 5 H**).^67^

## Surgery

Mice were fixed on the operating table after anesthesia. For vagotomy, the stomach was pulled out, and the vagus nerves on either side of the esophagus above the cardia were cut off (**Supplementary Fig. 5A**). For intra-intestinal injection (**Supplementary Fig. 5B-C & Supplementary Fig. 5 F-G**), FITC-labeled hAβ42 (FA-42-T-1, Shanghai Genita Biotech), ^125^ I-labeled hAβ42, and oligomeric hAβ42 (O-hAβ42) were injected into the serosa of the colon at a dosage of 50 μg per mouse.

## Two-photon intravital imaging

FITC-labeled human Aβ42 was injected into the colon of 10-month-old WT mice, and hAβ42 was visualized with two-photon intravital imaging 2 hours later. Under anesthesia, the skin and part of the dura mater tissues of the mice were removed, and a small craniotomy (~1 mm × 1 mm) was made on the parietal lobe of the mice. The mice were transferred to a FVMPE-RS two-photon microscope (Olympus, Tokyo, Japan) to perform two-photon imaging.

## Immunofluorescence staining

The tissues were immersed in 4% paraformaldehyde solution for 1 day at 4°C. After dehydration in 30% sucrose, tissue sections were prepared with a sliding microtome (Leica, Wetzlar, Germany). Sections were permeabilized with 0.4% Triton X-100 in PBS (PBST), blocked with 1% BSA, incubated with primary antibodies and incubated with corresponding secondary antibodies sequentially. The slices were then embedded in an antifade mounting medium with DAPI (ZG1028, VECTASHIELD). Immunofluorescence images were captured using an Olympus confocal instrument.

## 16S rRNA sequencing

The total DNA in fecal samples was extracted utilizing the MN NucleoSpin 96 Soi Kit. To analyze the taxonomic composition of the bacterial community, the V3-V4 region of the 16S rRNA gene was selected for pyrosequencing. PCR amplification was performed in triplicate using the custom barcoded universal bacterial primers. The PCR products were pooled, confirmed, cleaned, and finally sequenced on an Illumina HiSeq platform according to the standard protocols from Frasergen (Wuhan, China).

## Fecal microbiota transplantation

The fresh feces from 12-month-old APP/PS1 mice (donors) and newly weaned WT mice (recipients) were collected in tubes, dissolved in ice-cold PBS at 10 mg/ml, sonicated at 20 w (JY92-IIN, scientz, China) for a short time simply to break the fecal pellet without releasing the fecal APP or Aβ (**Supplementary Fig. 5I**) and then centrifuged at 300 g for 5 min at 4°C. The supernatants were transferred to new tubes and stored at −80°C. The WT littermates were randomly divided into two groups after weaning and gavaged with fecal supernatant (200 μL per mouse) of 12-month-old APP/PS1 mice or themselves as control three times a week.

## Behavioral assessments

The spontaneous Y-maze was used to assess the spatial working and short-term memory; the novel object recognition used the innate exploratory behavior of mice to assess memory; and the Morris water maze was employed to assess the spatial working memory. The three behavioral tests were conducted following previous study.^18^

## Statistical analysis

Statistical data were analyzed using GraphPad Prism 9. All data were shown as mean ± standard error mean (SEM). The unpaired two-tailed Student’s t-test was used for two-group comparisons. A one-way ANOVA followed by Tukey’s post hoc test was performed for multiple group comparisons.

## Supplementary Material

Supplemental Material

## Data Availability

All data generated or analyzed during this study are included in this published article and its supplementary information files.

## List of abbreviations

Aβ: β-amyloid; AD: Alzheimer’s disease; WT: wild type; APP/PS1: amyloid precursor protein/presenilin 1; FMT: fecal microbiota transplantation; NFTs: neurofibrillary tangles; APP: amyloid precursor protein; BACE1: β-secretase; PS1: Presenilin 1; PS2: Presenilin 2; BBB: blood-brain barrier; GM: gut microbiota; GI: gastrointestinal.
