# Supplementary material for: Gut-derived β-amyloid: Likely a centerpiece of the gut–brain axis contributing to Alzheimer’s pathogenesis
Source: Gut Microbes. 2023 Jan 22;15(1):2167172. doi: 10.1080/19490976.2023.2167172 (PMC9872956; doi:10.1080/19490976.2023.2167172)
Supplement: Supplemental Material [file KGMI_A_2167172_SM0206.zip › supplementary figure.docx]

**Gut-derived β-amyloid: Likely a Centerpiece of the Gut-brain axis Contributing to Alzheimer’s Pathogenesis**

***Supplement 1***

Supplementary figure 1


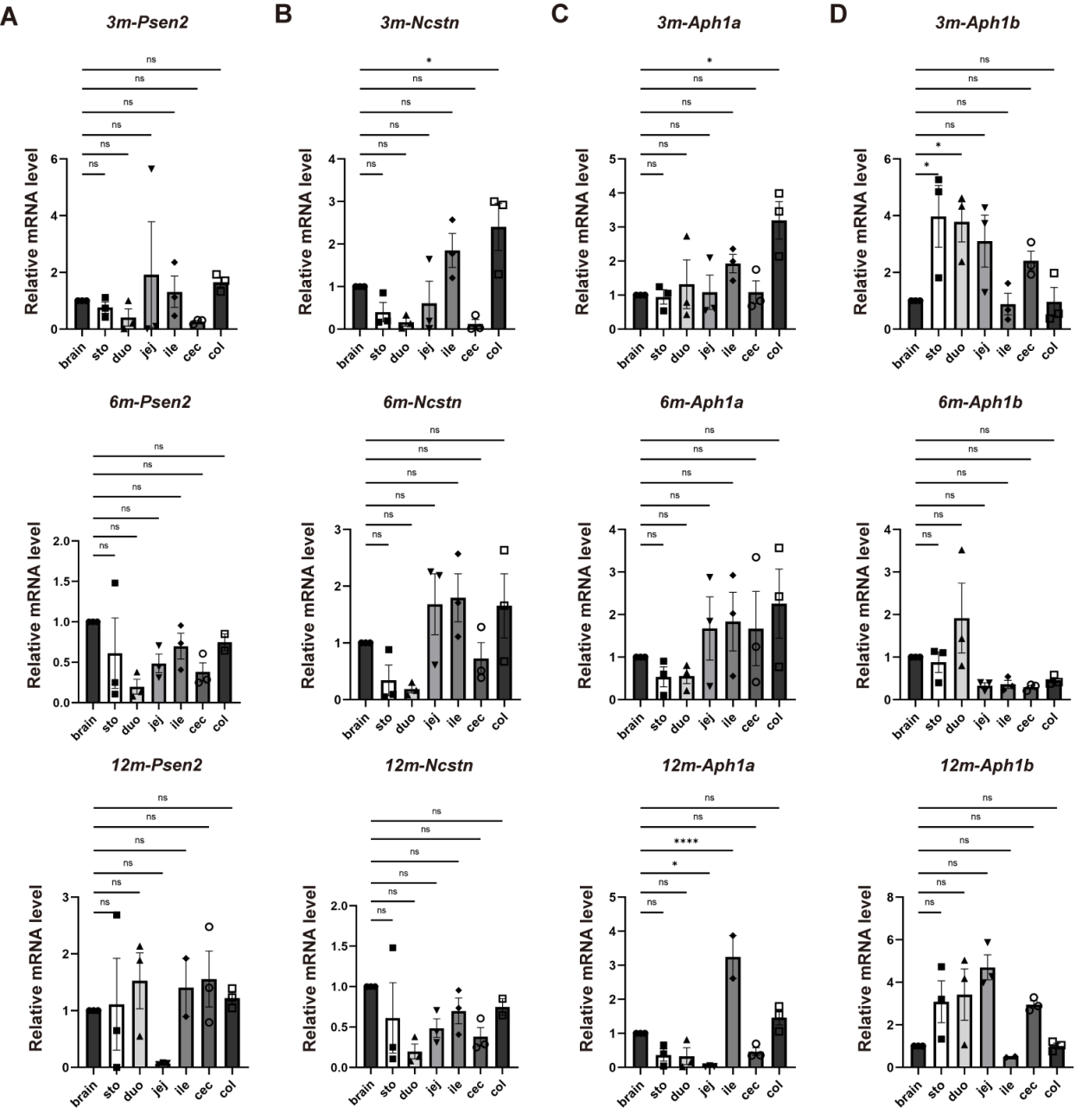


**Figure S1A-D.** Quantitative analysis of *Psen2*, *Ncstn*, *Aph1a,* and *Aph1b* mRNA levels in 3-, 6- and 12-month-old mice by qPCR (n = 3). The mRNA levels of genes in the stomach (sto), duodenum (duo), jejnum (jej), ileum (ile), cecum (cec) and colon (col) were compared with those in the brain for statistical significance. Values are means ± S.E.M, ordinary one-way ANOVA test. ns, no significance; *, P < 0.05; ****, P < 0.0001.

Supplementary figure 2


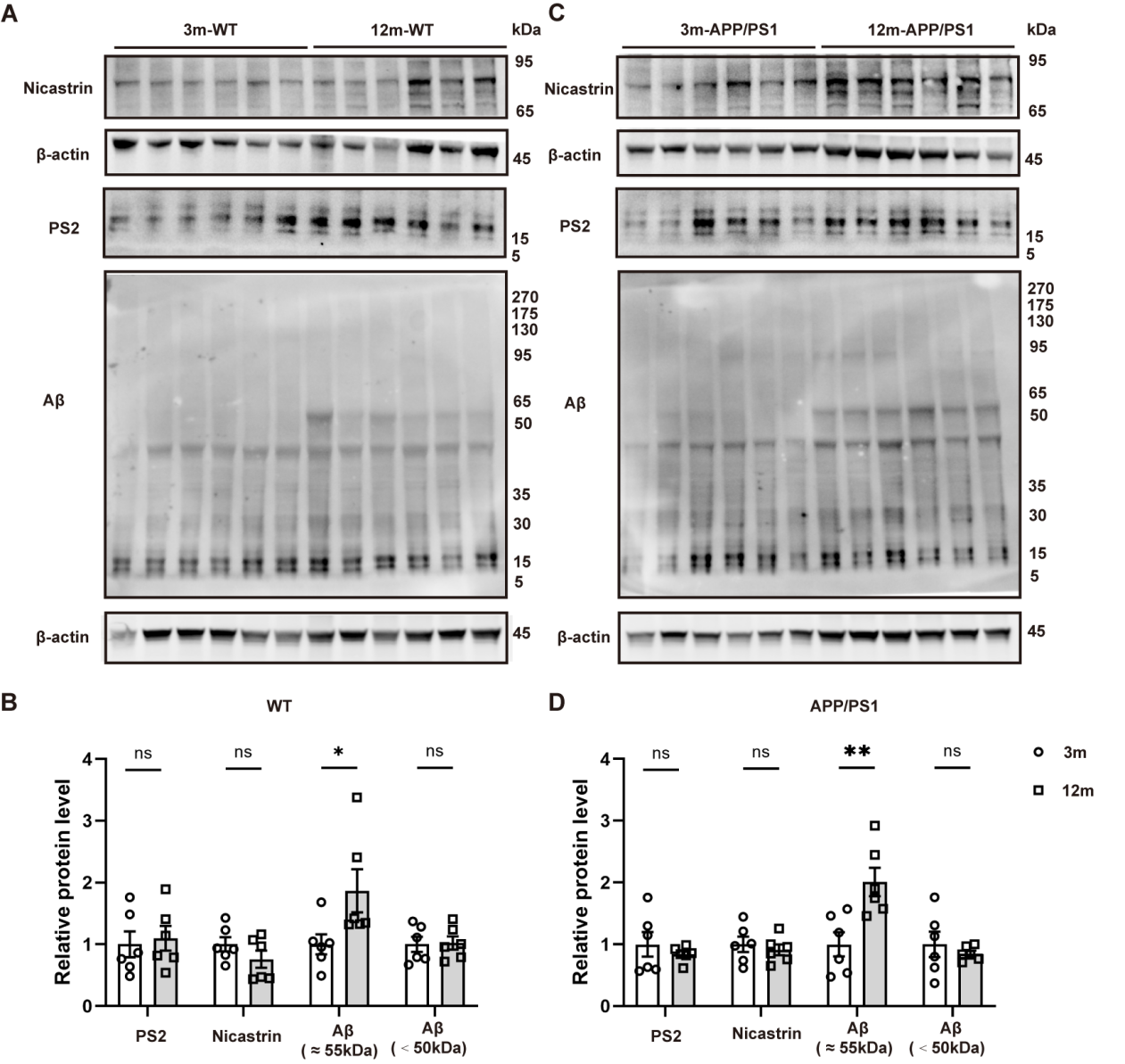


**Figure S2.** (**A-B**) WB images and quantitative analysis of colonic PS2 (Proteintech, 16168-1-AP), Nicastrin (Proteintech, 14071-1-AP) and Aβ (biolegend, 6E10, 803015) protein levels in WT mice aged 3 (n = 6) and 12 months (n = 6). (**C-D**) WB images and quantitative analysis of colonic PS2, Nicastrin, and Aβ protein levels in APP/PS1 mice aged 3 (n = 6) and 12 months (n = 6). For Aβ quantification, the oligomeric band (≈55kDa) and smaller bands ( < 50kDa) were seperately analyzed. Values are means ± S.E.M, unpaired t test. *, P < 0.05; **, P < 0.01.

Supplementary figure 3


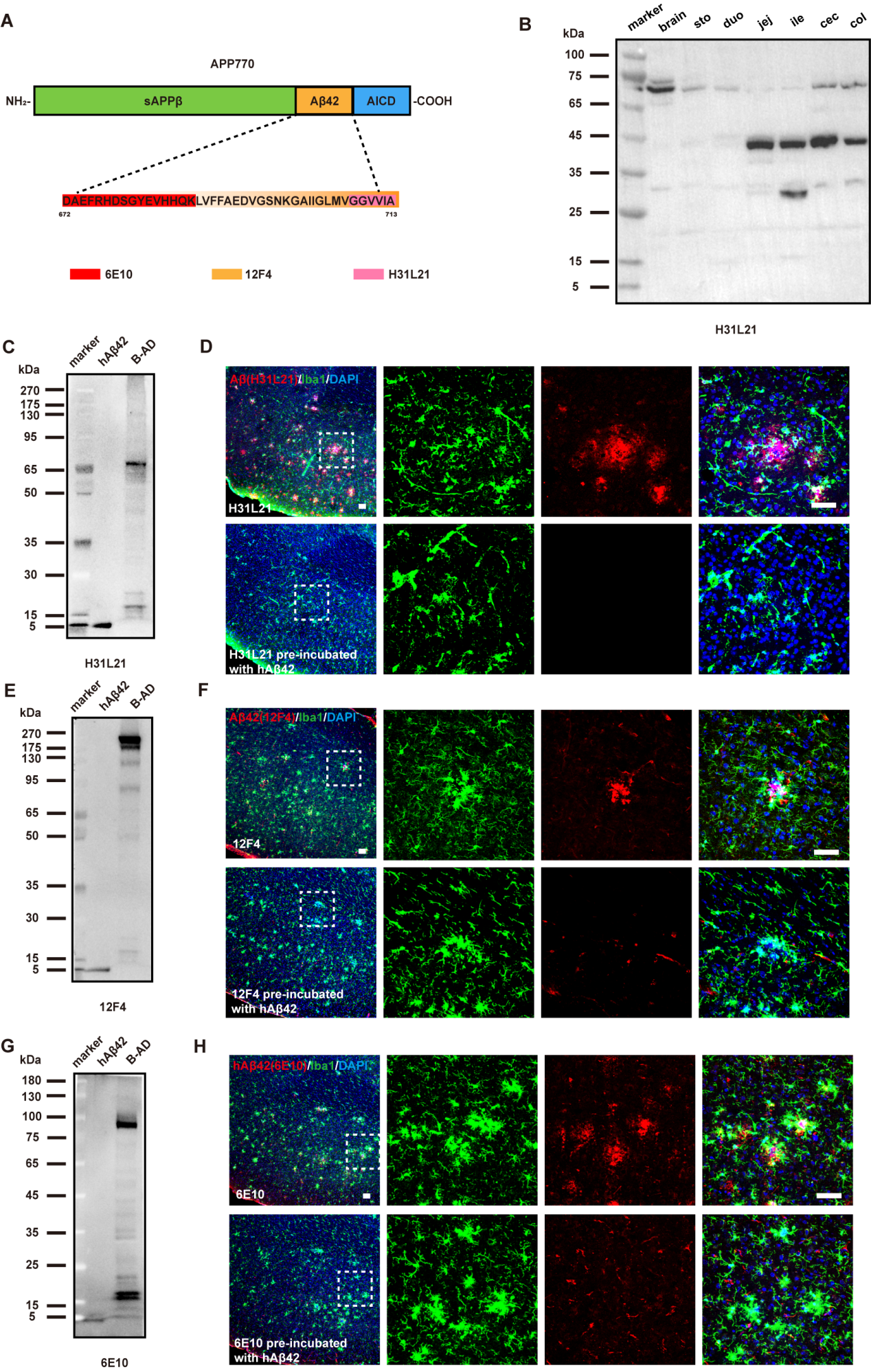


**Figure S3. (A)** Schematic diagram of the antigens of the antibodies used for the detection of APP/Aβ. **(B)** Full-length WB images of Aβ stained by H31L21 (Invitrogen, 700254) in the brain and gut of the WT mice. **(C)** Full length WB images of standard hAβ42 and brain of 20-month-old APP/PS1 mice (B-AD) stained by H31L21. **(D)** Confocal images of Aβ plaques stained by H31L21 or H31L21 pre-incubated with standard hAβ42 in the cortex of 20-month-old APP/PS1 mice. Scale bar = 50 μm. **(E)** Full length WB images of standard hAβ42 and B-AD stained by 12F4 (Biolegend, 805503). **(F)** Confocal images of Aβ plaques stained by 12F4 or 12F4 pre-incubated with standard hAβ42 in the cortex of 20-month-old APP/PS1 mice. Scale bar = 50 μm. **(G)** Full length WB images of standard hAβ42 and B-AD stained by 6E10 (Biolegend, 803015). **(H)** Confocal images of Aβ plaques stained by 6E10 or 6E10 pre-incubated with standard hAβ42 in the cortex of 20-month-old APP/PS1 mice. Scale bar = 50 μm.

Supplementary figure 4


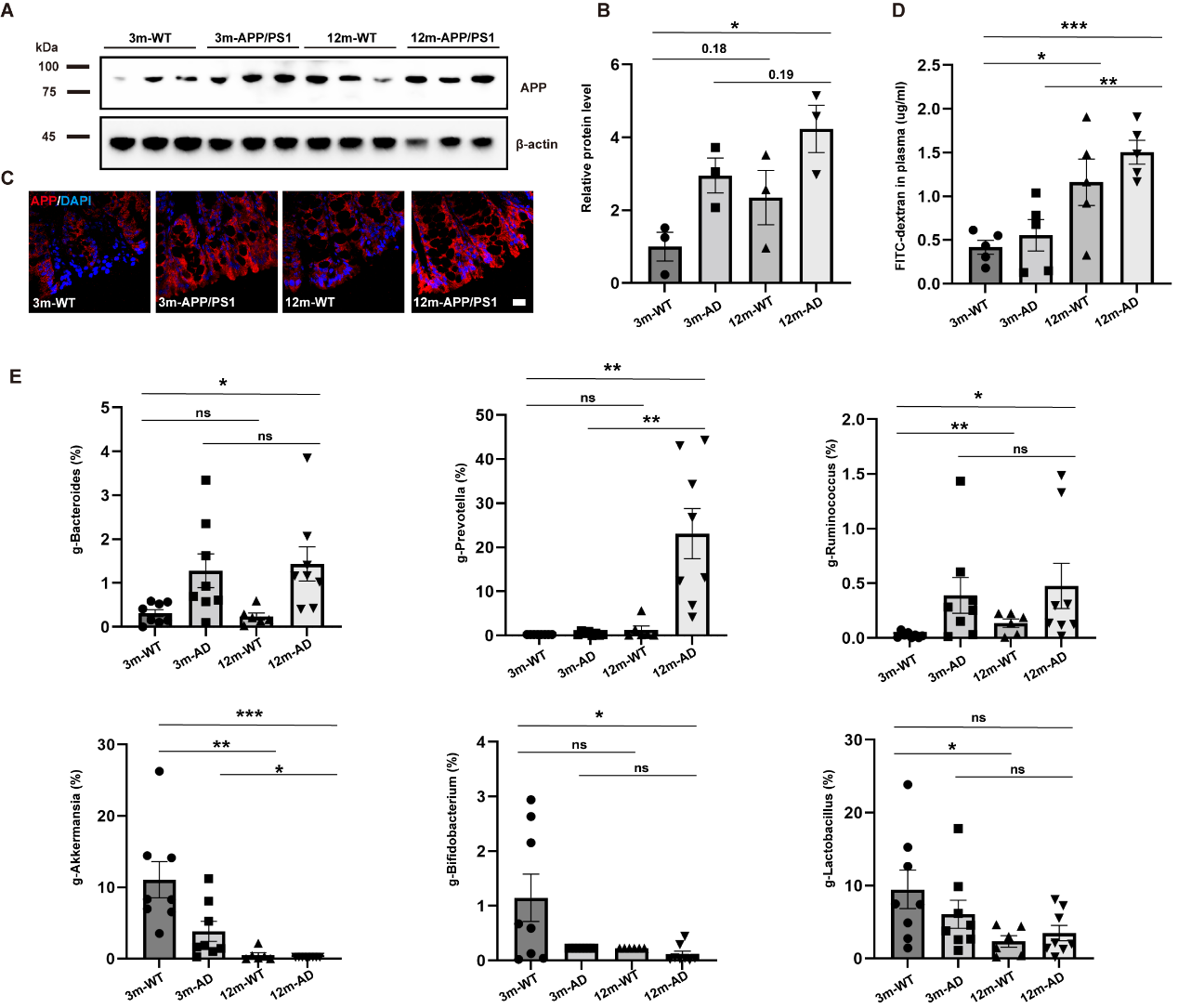


**Figure S4. (A-B)** WB images and quantitative analysis of APP stained by CT695 (Invitrigen, 51-2700) in the colon of WT and APP/PS1 mice aged 3 and 12 months old (n=3). **(C)** Confocal images of APP in the colon of WT and APP/PS1 mice aged 3 and 12 months old. Scale bar = 20 μm. **(D)** In vivo intestinal permeability as measured by the FITC-dextran assay in WT and APP/PS1 mice aged 3 and 12 months old. **(E)** Abundance of *Bacteroides*, *Prevotella*, *Ruminococcus*, *Akkermansia*, *Bifidobacterium*, and *Lactobacillus* based on 16S rRNA gene-sequencing dataset of feces from 3-month-old WT mice (3m-WT, n = 8), 3-month-old APP/PS1 mice (3m-AD, n = 8), 12-month-old WT mice (12m-WT, n = 6), and 12-month-old APP/PS1 mice (12m-AD, n = 8). Values are means ± S.E.M, unpaired t test. ns, not significant; *, P < 0.05; **, P < 0.01; ***, *P* < 0.001.

Supplementary figure 5
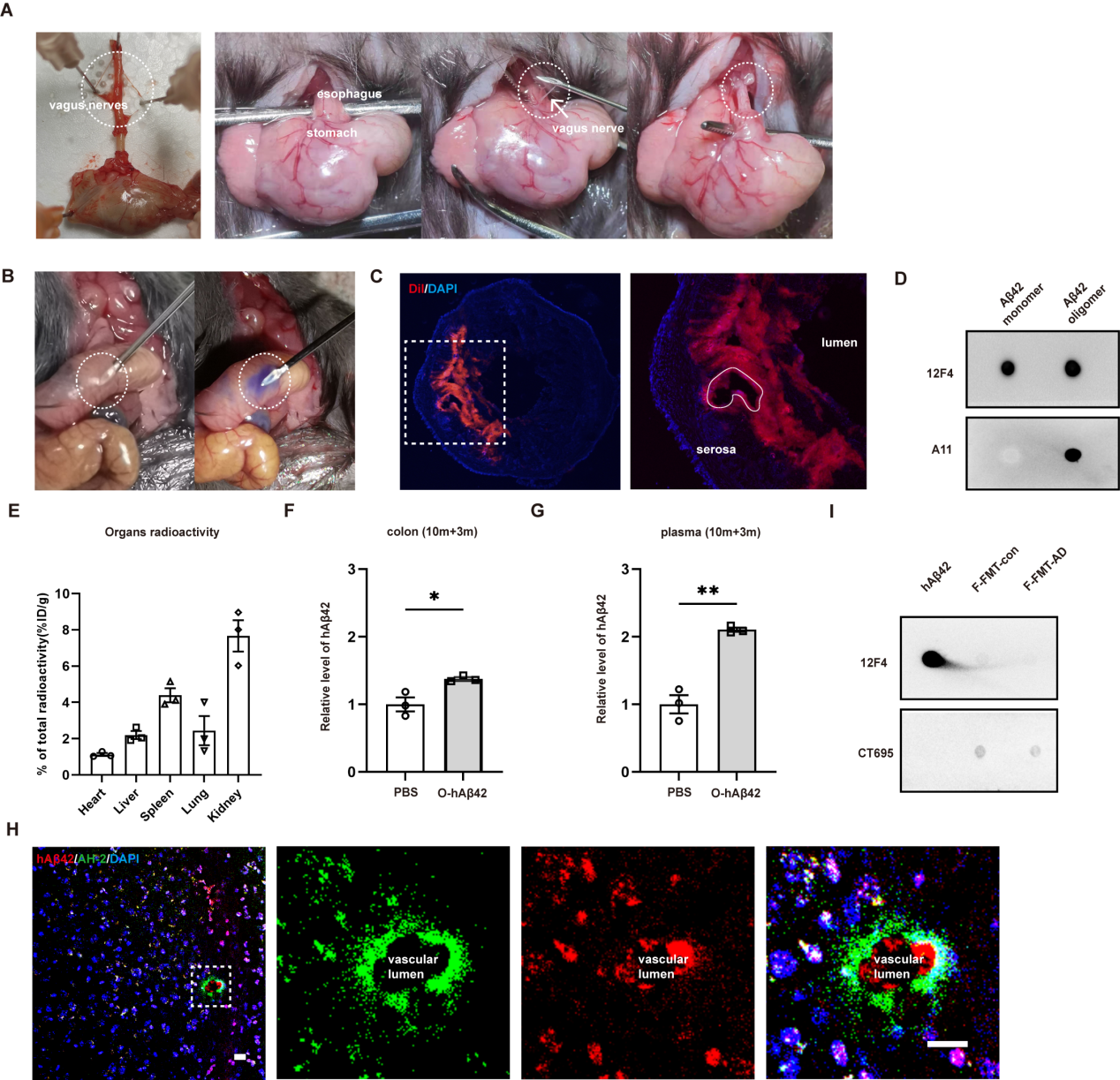


**Figure S5. (A)** Anatomical images of the vagus nerves on either side of the esophagus above the cardia and vagotomy images. **(B)** Intra-intestinal injected blue ink in the serosa of the colon of the WT mice. **(C)** Confocal images of the intra-intestinal injected DiI in the serosa of the colon of the WT mice. **(D)** Dot blot images of the A42 monomer and oligomer stained with 12F4 (Biolegend, 805503) and A11 (Invitrogen, AHB0052). **(E)** The radioactivity of heart, liver, spleen, lung, and kidney samples of 10-month-old mice after intra-intestinal injection of ^125^ I-labeled hAβ42. **(F & G)** ELISA quantitative analysis of hAβ42 in colon tissues and plasma 3 months after intra-intestinal injection of PBS or hAβ42 (O-hAβ42) oligomers in 10-month-old mice (10m+3m). **(H)** Confocal images of injected O-hAβ42 stained by 6E10 (Biolegend, 803015) and a homemade oligomer detection dye AH-2 in the blood vessels of the cortex in 10-month-old mice. Scale bar = 15 μm. **(I)** Dot blot images of the standard hAβ42, fecal supernatant gavaged to FMT-con (F-FMT-con), fecal supernatant gavaged to FMT-AD (F-FMT-AD) stained by 12F4 and CT695 (Invitrogen, 51-2700). Values are means ± S.E.M, unpaired t test. *, P < 0.05; **, P < 0.01.

**Full-length Western blots：**

**Fig 1F**

**
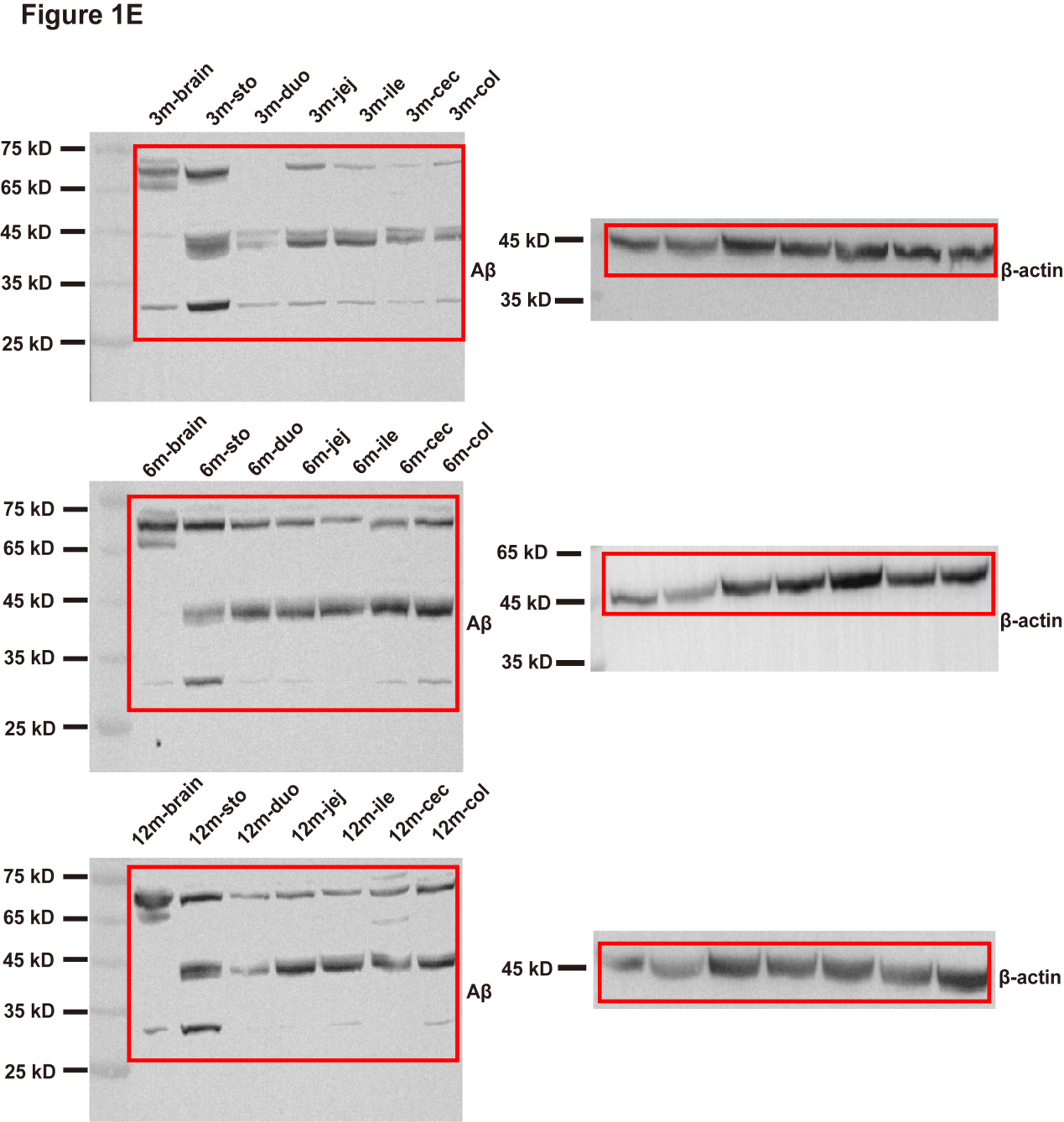
**

PVDF membranes were blotted with anti-Aβ [H31L21] (Invitrogen, 700254) and anti-β-actin, respectively. *Red boxes* indicate the cropped images shown in **Fig. 1F**.

**Fig 2A & C**


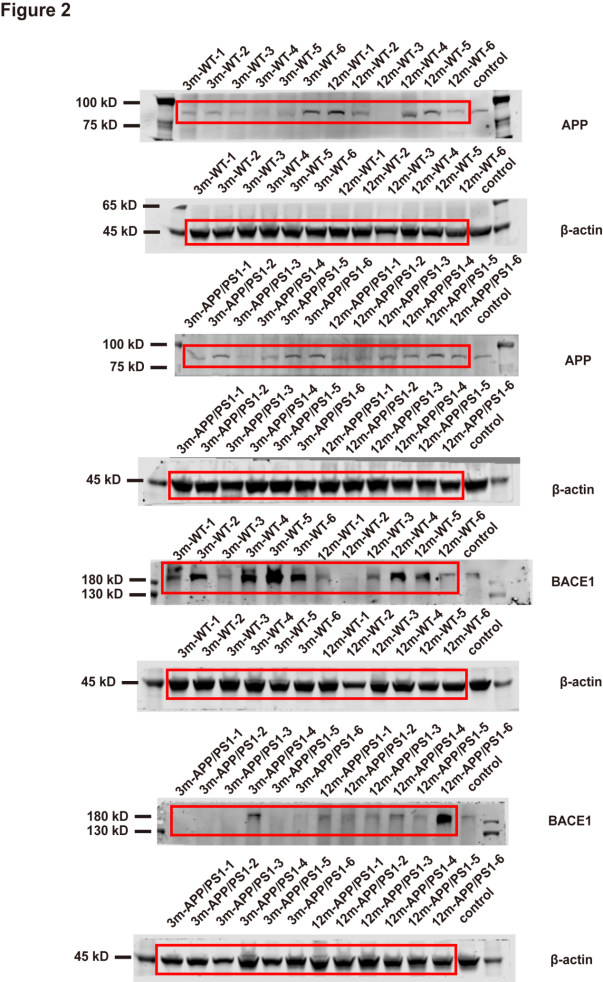

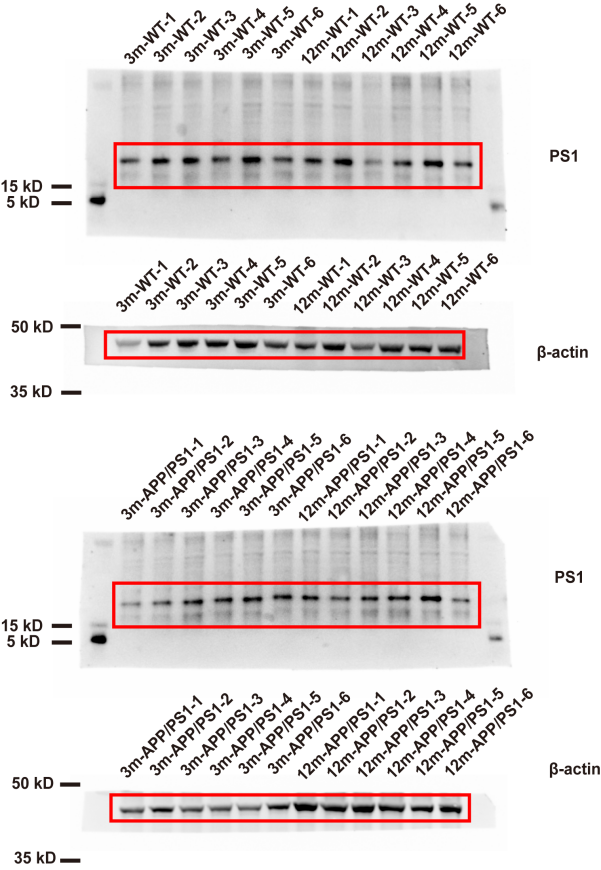


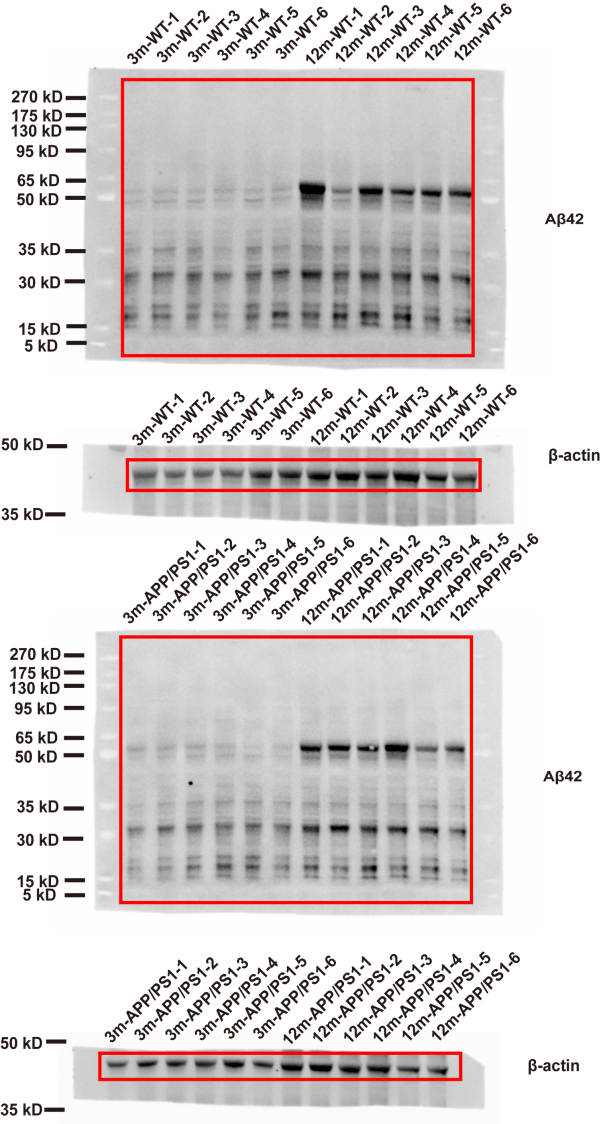


PVDF/NC membranes were blotted with anti-APP [CT695] (Invitrogen, 51-2700), anti-BACE1 (Invitrogen, PA1-757), anti-PS1 (Abcam, ab76083), anti-Aβ42 [12F4] (Biolegend, 805503) and anti-β-actin, respectively. For quantification of APP, BACE1 and PS1, strips blotted with β-actin were from the same gel/membrane. For quantification of Aβ, the same gel/membranes were first blotted with 12F4, stripped and then blotted with anti-β-actin. *Red boxes* indicate the cropped images shown in **Fig. 2A & C**.

**Supplementary Fig. 2**


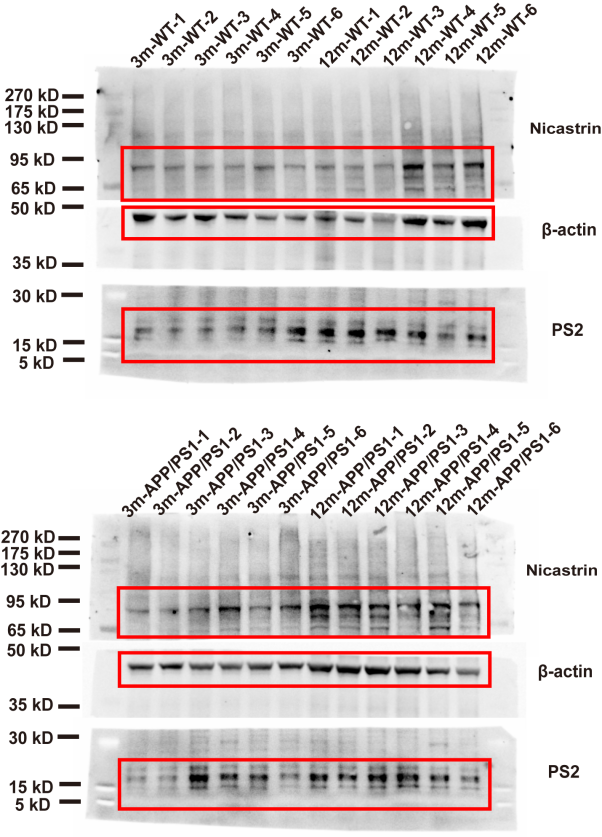

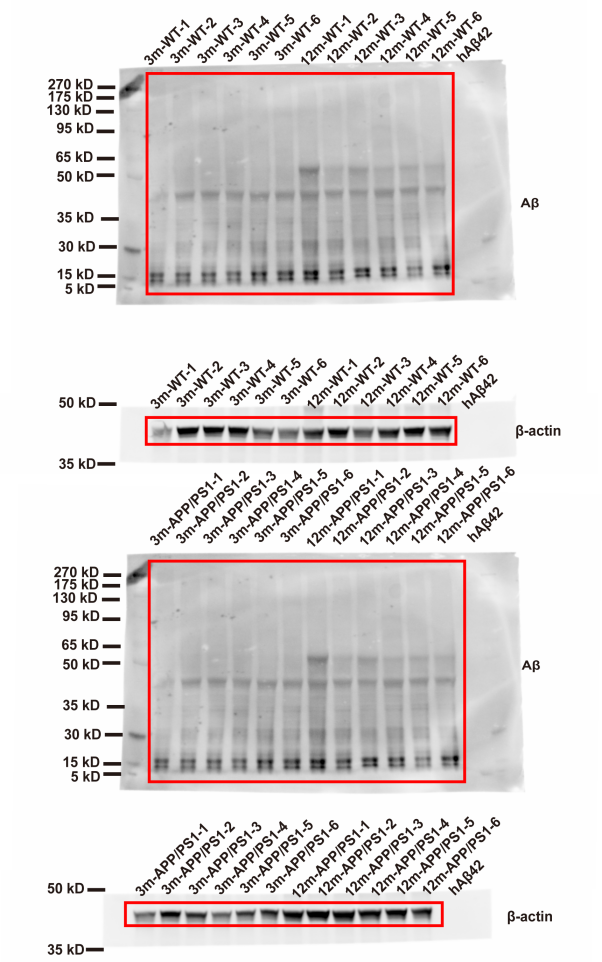


PVDF/NC membranes were blotted with anti-Nicastrin (Proteintech, 14071-1-AP), anti-PS2 (Proteintech, 16168-1-AP), anti-Aβ [6E10] (Biolegend, 803015) and anti-β-actin, respectively. For quantification of Nicastrin and PS2, the same PVDF/NC membranes were separated into three strips. *Red boxes* indicate the cropped images shown in **Supplementary Fig. 2**.


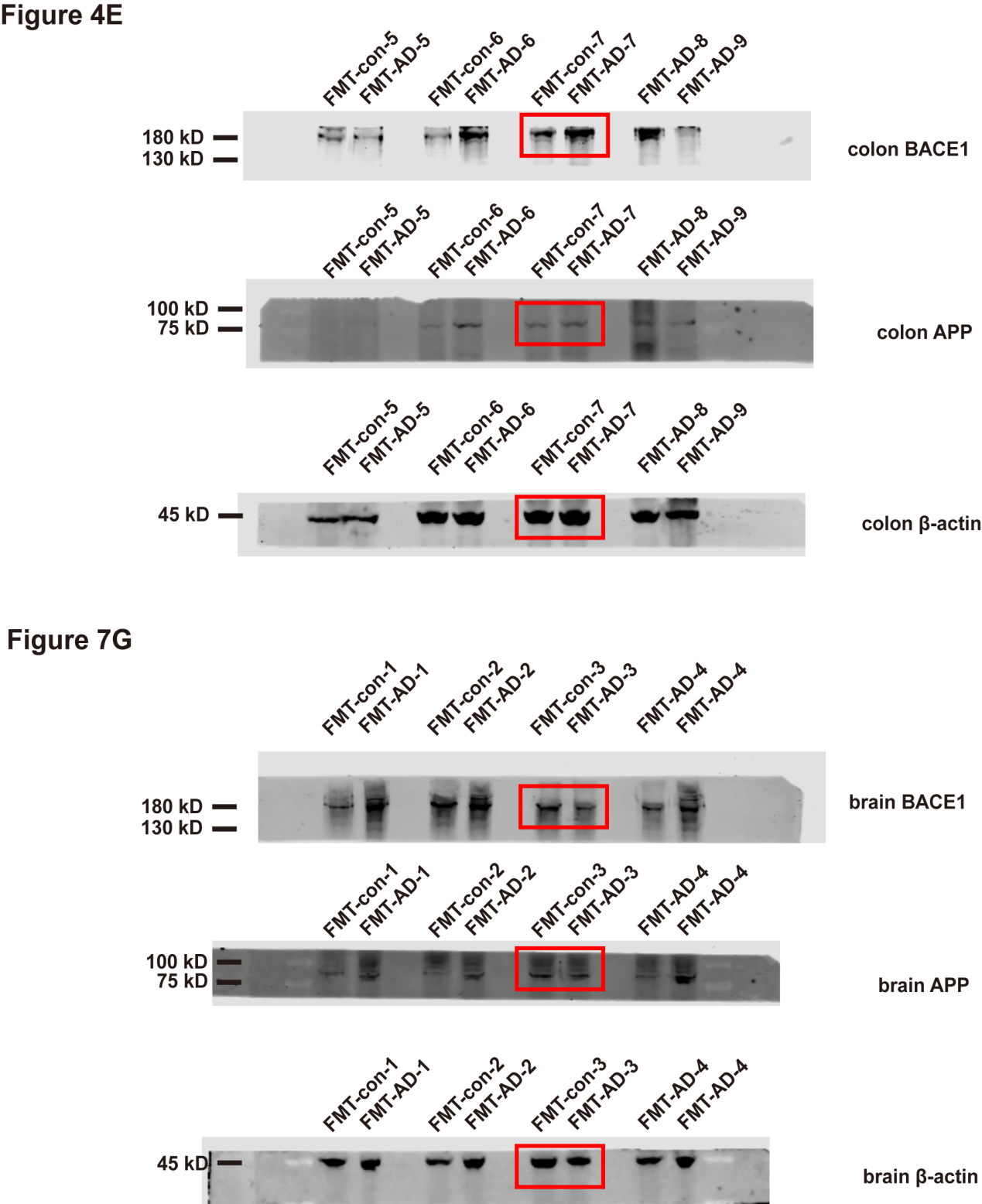


PVDF membranes were separated into three strips for blotting with anti-BACE1, anti-APP and anti-β-actin, respectively. *Red boxes* indicate the cropped images shown in **Fig. 4E**.


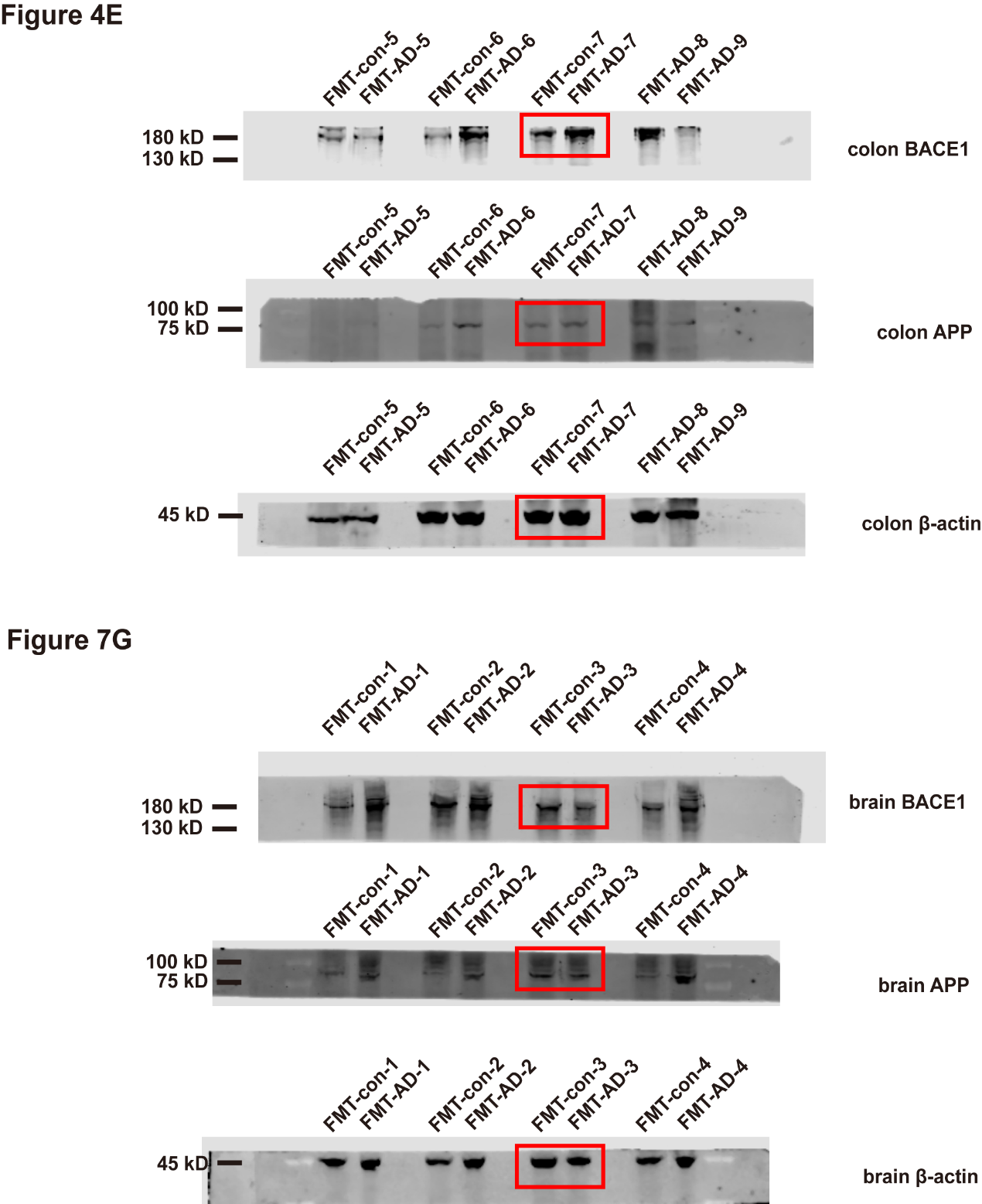


PVDF membranes were separated into three strips for blotting with anti-BACE1, anti-APP and anti-β-actin, respectively. *Red boxes* indicate the cropped images shown in **Fig. 7G**.

**SUPPLEMENTAL TABLE TLTLES**

**Table S1. Demographics and clinical data of patients.**

**Table S2. The primers for mRNAs.**

***See Supplement 2 (Excel file) for all supplemental tables.***
